# Supplementary material for: A Global Screen for Assembly State Changes of the Mitotic Proteome by SEC-SWATH-MS
Source: Cell Syst. 2020 Feb 26;10(2):133–155.e6. doi: 10.1016/j.cels.2020.01.001 (PMC7042714; doi:10.1016/j.cels.2020.01.001)

O75400 | PR40A_HUMAN | PRPF40A FBP11 FLAF1 FNBP3 HIP10 HYPA HSPC225
Monomer MW [kDa]: 108.805 Monomer expected elution fraction: 39

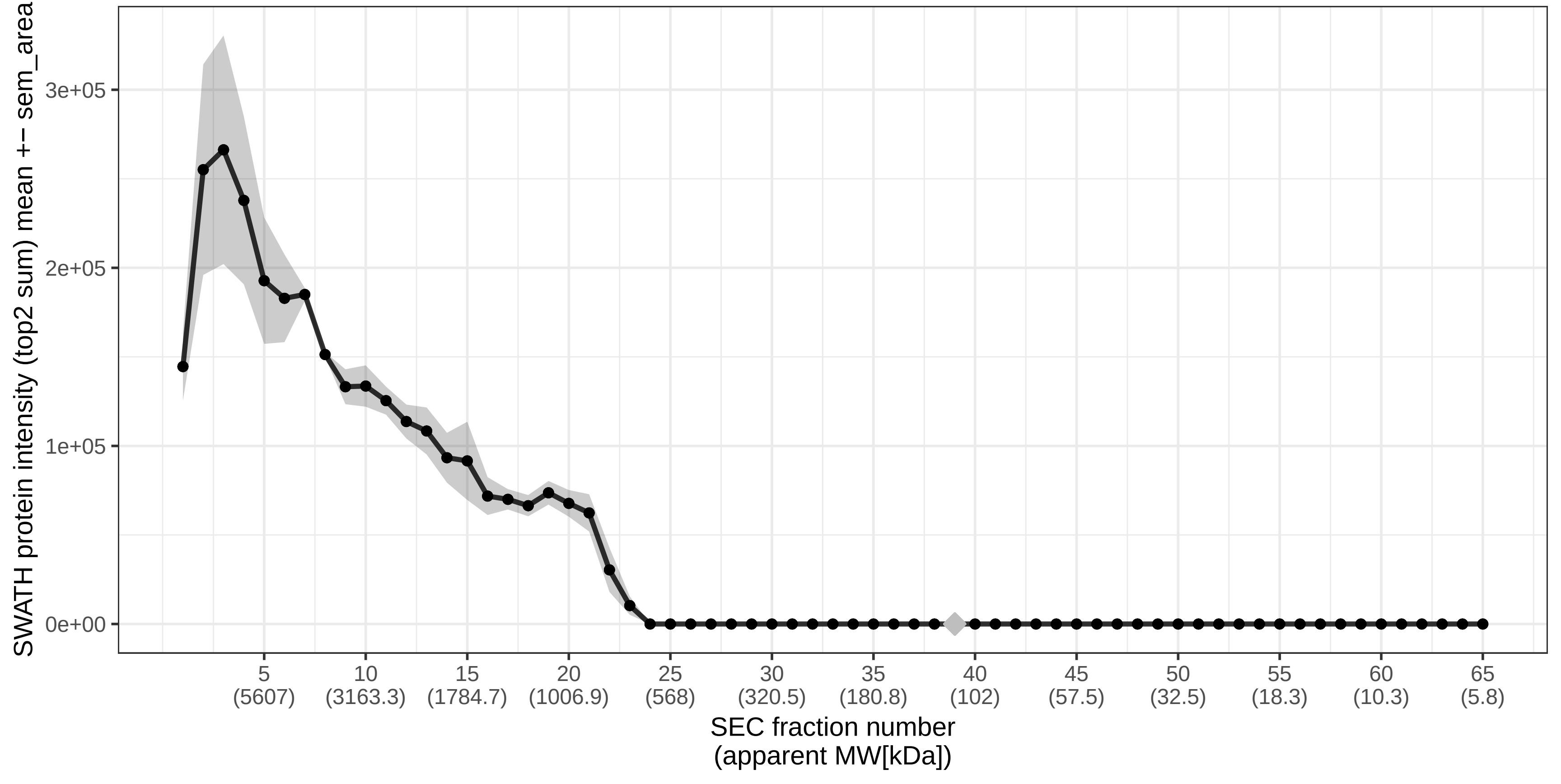

Supplement: Data S1. SEC-SWATH-MS Protein Chromatograms, Related to Figure 1 [file mmc6.zip › SECchrom_O75400_PR40A_HUMAN_PRPF40A_FBP11_FLAF1_FNBP3_HIP10.pdf]
